# Intelligent Assessment Techniques for Abnormal Movement Patterns in Neurological Disorders: Applications and Advances

**DOI:** 10.1155/bn/6006064

**Published:** 2025-10-05

**Authors:** Yunjun Bao, Ronghua Hong, Wenting Qin, Zhuang Wu, Yunping Song, Lingjing Jin

**Affiliations:** ^1^Department of Neurology and Neurological Rehabilitation, Shanghai Disabled Persons' Federation Key Laboratory of Intelligent Rehabilitation Assistive Devices and Technologies, Yangzhi Rehabilitation Hospital (Shanghai Sunshine Rehabilitation Center), School of Medicine, Tongji University, Shanghai, China; ^2^Neurotoxin Research Center, Key Laboratory of Spine and Spinal Cord Injury Repair and Regeneration of Ministry of Education, Department of Neurology, Tongji Hospital, School of Medicine, Tongji University, Shanghai, China; ^3^Collaborative Innovation Center for Brain Science, Tongji University, Shanghai, China

**Keywords:** abnormal movements, intelligent assessment, movement analysis, neurological disorders

## Abstract

Neurological disorders frequently result in diverse forms of abnormal movement. Conventional clinical assessment approaches often lack the precision and objectivity needed to evaluate muscle involvement and associated functional limitations. With the development of various intelligent assessment devices, technologies such as wearable sensors, motion capture, radar, and imaging technology, which are based on myoelectricity, kinematics, mechanics, and optics, combined with mathematical models and algorithms, have been widely used for abnormal movement recognition. These technologies further improve the accuracy and validity of clinical evaluation. In this paper, we review the latest advances in intelligent technologies that help clinicians qualitatively and quantitatively assess abnormal movement patterns and carry out personalized rehabilitation treatment. Our work was also aimed at summarizing the research and application of intelligent assessment techniques.

## 1. Introduction

Patients suffering from neurological conditions frequently display abnormal motor patterns, characterized by deviations from intended movement trajectories due to disrupted muscle coordination. Such patterns often involve synergistic movements of the entire limb with reduced independent joint control [1]. In the context of functional movement disorders, these abnormalities are clinically categorized according to their phenomenological presentation, including tremor, myoclonus, gait disturbances, parkinsonism, and tics. Many patients with neurological disorders, such as stroke, spinal cord injury, multiple sclerosis, cerebral palsy, and peripheral nerve injury, can develop abnormal movements. Abnormal movements limit limb movement, fine motor skills, and overall mobility, resulting in complications such as joint contractures and pain, which further exacerbate the impairment of motor function [2–4].

Clinical scales are common tools for assessing abnormal movements, which are based on the observation of the patient's motor performance by clinicians [5]. Although scale-based assessment methods do not require sophisticated equipment and are clinically feasible, they are subjective and cannot objectively reflect muscle activity during dynamic tasks (e.g., gait). Moreover, the use of clinical scales usually results in difficulty in capturing subtle movements, which sometimes presents obstacles in differential diagnosis [6–8]. For abnormal gait, scales like the Tinetti Gait Scale, 6-min Walk Test, and Time Up and Go cannot adequately assess disease severity or specific characteristics like stability and asymmetry [9]. Dystonia assessments (e.g., Modified Ashworth Scale and Tardieu Scale) fail to examine the underlying muscle contraction patterns or identify target muscles [10]. Tremor rating scales (e.g., Fahn-Tolosa-Marin Scale) lack specificity for conditions like PD and cannot accurately quantify frequency or aid differential diagnosis [11]. Parkinsonism relies heavily on the Unified Parkinson's Disease Rating Scale III, which is insufficient for tracking severity and progression. Myoclonus scales (e.g., Unified Myoclonus Rating Scale) are prone to inaccuracy due to variable influencing factors and are time-consuming [12].

With the development of modern measurement techniques, electronics and computer expertise, intelligent instruments have been applied for the qualitative and quantitative assessment of abnormal movements. Current intelligent assessment techniques are based on motion capture and kinetic analysis technologies such as optical motion capture (OMC), markerless vision-based motion capture, radar technology, and inertial sensor technology, as well as robotic feedback technology, to accurately track and analyze abnormal movements. Additionally, physiological and neuromuscular technologies such as multilead electromyography (EMG), ultrasound technology, infrared thermography, and positron emission tomography (PET)/single-photon emission computed tomography (SPECT) muscle metabolism imaging provide valuable insights into muscle and neural activity, contributing to a more comprehensive understanding of abnormal movements.

This paper reviews recent assessment devices, highlights identifiable abnormal movement patterns, and summarizes intelligent methods for distinguishing their severity. These may hold potential for aiding clinicians and therapists in developing rehabilitation plans.

## 2. Search Strategy and Selection Criteria

**Figure 1:**
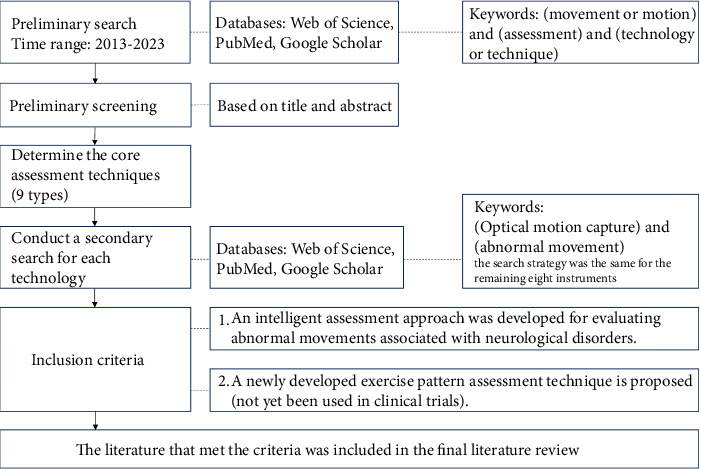
Flowchart of literature retrieval.

A literature review was performed using the keywords “(movement or motion) and (assessment) and (technology or technique)” on Web of Science, PubMed, and Google Scholar on September 2, 2023. The search criteria were studies that were published from 2013 to 2023. After the abstracts of the selected studies were read, nine abnormal motion pattern assessment instruments were selected, and the literature search was conducted again. The keywords were “(Optical motion capture) and (abnormal movement),” and the search strategy was the same for the remaining eight instruments. The inclusion criteria were studies that presented an intelligent assessment method for the assessment of abnormal movements caused by neurological diseases and studies that presented newly developed techniques for assessing movement patterns but that have not yet been used in clinical trials (Figure 1).

## 3. Motion Capture and Kinetic Analysis Technologies

### 3.1. OMC

#### 3.1.1. Mechanism

The optical motion capture system (OMCS) requires multiple infrared cameras to track infrared-illuminated marker points attached to key body parts from different angles. It depends on the reflective markers to reflect the infrared light from the infrared cameras, after which the 3D coordinates of the markers, which are based on the coordinates of the reflected light captured by the cameras, are calculated. It is used for accurate measurement of 3D kinematic information of the human body in indoor environments [13]. The OMC is a widely accepted standard for kinematics estimation, but due to the high cost of the equipment and the need for preplanned space conditions, its use is limited. Multicamera systems need to be installed and calibrated in the laboratory before use, and skin-based markers might not accurately depict the underlying bone movement, a source of inaccuracy known as soft tissue artefacts, which cause poor accuracy in assessing wrist movements [14]. Optical marker-based motion capture is therefore limited in terms of practical clinical applications.

#### 3.1.2. New Findings

Recent advances in OMC have introduced several cost-effective and accessible alternatives to traditional gait and limb tracking systems. VICON is the world's first OMCS. One approach combined the Vicon Nexus system with machine learning (ML) to automate gait analysis [15]. Wearable skin markers detectable by conventional RGB cameras have been developed for low-cost and accurate motion analysis [16]. This technique enables the analysis of six-degree-of-freedom movements of human segments and is comparable to the accuracy of VICON-based systems in joint angle calculation, except for left and right hip rotation. Therefore, it can be used in the field of home gait analysis and has wide application prospects. Wang et al. [17] developed a gait recognition method that uses 10 motion trackers to track lower body positions. A kernel extreme learning machine classifier was used to recognize the gait process for single-frame motion data. This method can achieve 100% verification accuracy with fewer than 50 frames of gait movement. In hand tracking, the Leap Motion Optical Hand Tracking Controller uses two infrared LED emitters and three infrared cameras to capture hand movements and has been identified as suitable for medical needs. When integrated with the AnyBody Modeling System (a musculoskeletal software simulation solution using inverse dynamics analysis), it offers a fast and low-cost solution for simulating forearm motion in biomechanical assess [18].

#### 3.1.3. Disease Identification and Severity Assessment

OMC has been used in stroke and PD patients to distinguish the severity of the disease and compared stroke patients with healthy individuals to identify subtle patterns that are invisible to the eye. OMC has proven to be valuable in analyzing gait [19]. The Vicon 370 motion capture system has been applied to study the patterns of mechanical energies of lower limb segments [20]. Researchers have reported that the kinetic energy in the thigh and shank of stroke survivors is negligible compared with that of healthy adults at a slow speed. Compensatory strategies emerge in stroke survivors during the swing phase. Beyond gait, Xie et al. [21] used Microsoft Azure Kinect and Leap Motion to evaluate compensatory strategies involving the upper limb and hand for stroke patients. They found that patients with stroke showed greater trunk rotation; greater extension of the metacarpophalangeal and proximal interphalangeal joints during pen grasp; a greater range of motion (ROM) of the metacarpophalangeal joint of the middle finger and the proximal interphalangeal joints of the middle, ring, and little fingers; and inadequate flexion in the index finger at the end of the grasp. This method can distinguish healthy individuals from stroke patients but cannot assess the severity of stroke. In upper limb assessments, an 8-camera 3D motion capture system was utilized to record upper body kinematics during the finger-to-nose test [22]. They showed how finger-to-nose test distinguishes between patients with mild and moderate upper limb deficits, and they identified the two factors most closely associated with stroke severity: time and elbow flexion during the pointing phase. Moreover, the patients in the stroke group moved their scapula and trunk excessively and with less accuracy than the controls did at similar speeds. In PD assessment, Leap Motion has demonstrated the ability to evaluate the severity of hand tremors and showed that maximal tremor movements can be precisely captured at the distal position of the wrist or hand by monitoring metrics, including volume, acceleration, and velocity [23].

### 3.2. Unmarked Vision-Based Motion Capture Systems

#### 3.2.1. Mechanism

Visual motion capture systems, which include conventional RGB cameras and depth cameras, acquire kinematic information remotely through markerless motion capture using camera equipment. The feature extraction algorithm is employed to extract the detected information with wave signal features that correspond to specific actions and transmit it to a computer terminal. Computer vision (CV) technology is then utilized to automatically analyze the video, extract information, and rapidly provide feedback, which results in highly precise motion capture and analysis [24]. Unlike traditional marker-based motion capture systems, markerless motion capture systems do not require marker points to be placed on the captured body, thus avoiding the interference and restriction of motion by marker points [25]. CV is a technology that uses computer and mathematical methods to analyze and process digital images or videos to automatically understand and analyze visual information [26]. It can detect abnormal patterns by recognizing and analyzing the postures of the human body. Deep learning (DL) and ML are used to train computers to recognize normal and abnormal poses by feeding images or videos into a trained model for estimating and recognizing human poses, joint centers, and bone positions. Abnormal patterns can be detected through a comparison of the difference between the actual pose and the normal pose. DL has a deeper neural network model architecture with ideas derived from the biological neural network of the human brain. The camera shooting distance and sampling frequency of video recording have little influence on the accuracy of the DL algorithm [27].

#### 3.2.2. New Findings

DL-based methods have recently been used to automatically estimate human joint centers. By capturing 2D joint positions from multiple simultaneous camera views and applying DL algorithms, joint centers and bony landmarks can be localized in 3D space [28]. Zhao et al. [29] proposed a contactless approach for identifying aberrant gait behavior. It uses a monocular camera to record human pose information. Using depth-wise separable convolution, a lightweight OpenPose model was created to identify joint points and extract their coordinates in real time while walking, and it was able to retrieve 11 different types of aberrant gait characteristics. The random forest (RF) method, when combined with 3D features, achieved the highest precision (92.13%) for recognizing aberrant gait behavior. The OpenPose human pose recognition project is the world's first real-time multiperson 2D pose estimation application based on DL that enables the evaluation of human movements, facial expressions, finger movements, and more. The convolutional neural network (CNN) algorithm is more commonly used because it has the shortest overall computation time and the least amount of computation for the same hardware conditions. The scheme overcomes the challenges of data drift and sensor wear from inertial sensors while decreasing the hardware needed for model deployment. The scheme has good real-time and noncontact properties and is expected to gain wide application in the field of anomalous gait assessment. Using an unmarked CV single-camera smartphone application based on Google Pose Estimation BlazePose, Young et al. [30] performed a running gait assessment. They extracted the contact time, swing time, stride length, knee flexion angle, and foot strike position from a large number of runners and used the gold standard Vicon 3D motion capture system as a reference. The assessment solution showed good consistency, offering a new approach to gait assessment in everyday environments. Kim and Neville [31] created a CV-based three-dimensional motion capture system. They used two action cameras to analyze the dexterity of hand movements while manipulating an object. To identify improvements in fine hand movements following practice, this model assesses the endpoint kinematics of goal-directed arm-reaching actions. This system should be tested for reliability and validity in patient groups in the future and is expected to be used to assess the effects of Parkinson's tremor on fine hand movements. When assessing upper limb horizontal extension tasks, the Kinect had better reliability when measuring trunk forward compensation and was insensitive to low-amplitude trunk rotation. Reliability was poor when variables such as elbow extension, shoulder flexion, and shoulder abduction were assessed [32].

#### 3.2.3. Disease Identification and Severity Assessment

The Kinect consists of a depth camera and an infrared camera-based motion capture system that enables real-time tracking of joint movements throughout the body [33, 34]. Studies have shown it accurately quantifies upper limb kinematics in stroke patients [35]. Azure Kinect can be used to track the clinical progress of PD patients. The system currently enables the automatic grading of the severity of trunk postural anomalies in PD patients, with a human–machine agreement of up to 0.940. The results revealed that F7 (F7: distance between the most convex point of the back and the trunk axis, the pixel distance between the FC [vertebral fulcrum] and the connecting line of C7 [the seventh cervical spinous process] and L5 [the fifth lumbar spinous process] on the sagittal plane) serves as a crucial metric for assessing the severity of camptocormia [36, 37]. Kinematic parameters such as step speed, stride length, sitting speed, and foot lift height allow objective evaluation of PD motor symptoms under medication [38]. Hong et al. [36] proposed a summary index for postural abnormalities (IPA) based on a Kinect depth camera. The findings demonstrated that the IPA had a high AUC (area under the curve) of 0.999 for distinguishing PD patients from healthy controls and an AUC of 0.817 for distinguishing PD patients with postural abnormalities from PD patients without postural abnormalities. Theia3D is a cost-effective markerless motion capture method that uses deep CNN and multiple 2D cameras to track human movement patterns. One study compared the accuracy of Theia3D to that of a marker-based method to analyze the gait of patients with cerebral palsy and chronic stroke [39]. The two methods yielded similar results, demonstrating the feasibility of using this markerless technique in a clinical setting. Camera-based gait monitoring systems may be affected by lighting conditions and clothing. The viewing angles are limited to approximately 60°, the maximum measurable distance is only a few meters, and privacy is often not protected. Therefore, this system may not be accepted as a home model [40]. Hand posture evaluation via CV involves locating anatomical hand keypoints in videos to measure joint ROM, aiding hand rehabilitation [41]. It also differentiates voluntary movements from tremors in PD [42].

Recent CV-based approaches used to assess neurological conditions are shown in Table 1. These methods leverage techniques such as Eulerian video magnification, ML classifiers (e.g., support vector machine [SVM]), and keypoint-based motion tracking to capture subtle motor features including tremor amplitude, postural control, and rhythmicity of finger tapping. The studies demonstrate the feasibility and accuracy of noncontact assessments, offering promising alternatives to traditional clinical tools.

### 3.3. Radar Technology

#### 3.3.1. Mechanism

Radar can extract echo signals generated by small movements of the human body to derive characteristic parameters of human motion, such as walking or running speed, leg swing frequency, arm swing frequency, arm swing amplitude, and other time delays [46]. These characteristic parameters can be used to recognize and classify movement patterns for gait assessment. Radar-based gait sensing is an emerging technology that has the advantages of being light independent, nonwearable, highly accurate, and having a long measurement distance. It plays an important role in human gait pattern recognition [47].

#### 3.3.2. New Findings

A Doppler radar system was shown to reliably extract five spatiotemporal gait parameters related to cadence and pace, demonstrating its applicability in gait analysis. This was confirmed through a quantitative comparison of measured biomechanical parameters with those obtained from motion capture and ground reaction forces. It was a new method to obtain the velocity of individual lower limb joints [47]. A radar micro-Doppler–based home gait analysis method was proposed by Seifert et al. [48], and they concluded that the radar micro-Doppler signal combined with the Fourier transform is most suitable for capturing gait changes, but the limited micro-Doppler resolution makes hinders precise identification. Jiang et al. [49] developed an innovative gait classification method based on millimeter wave array radar technology. They used a multichannel 3D CNN to extract motion features and classify typical daily movements. This method can achieve an accuracy of over 92.5% in recognizing common gait patterns, such as running and walking. Soubra et al. [50] used Doppler radar system to automate the TUG test and analyze gait and balance, which can identify early mobility decline.

#### 3.3.3. Disease Identification and Severity Assessment

Pulse radio ultrawideband (IR-UWB) radar sensors have been developed to detect objects without interference by using ultrawideband frequencies. The main advantages of their clinical application are their low power and high spatial resolution, which facilitate their ability to detect fine movements of objects and diagnose subjects in a noncontact manner without inconvenience to the patient. Because of its excellent penetration, it can be mounted covertly on walls and can be observed without drawing the attention of the target [51]. Thus, IR-UWB radar sensors hold great promise for the evaluation and quantification of clinically abnormal motion patterns. Yim et al. [52] showed that the IR-UWB radar sensor can be used as a clinical aid in the assessment of movement disorders. Two new measurements were presented that are specific to the activity—with or without location movement—with movement—with the signal strength and distance value data, which can potentially be applied in measuring the activity in movement disorders. Na et al. [53] used IR-UWB radar to quantitatively assess left–right motor asymmetry in infants, demonstrating that IR-UWB radar can be a new early screening tool for infants with developmental delays. UWB technology combined with SVM algorithms has also been used to detect gait abnormalities in patients with ataxia at home [54]. Currently, most radar research remains at the prototype or small-sample experimental stage and has yet to undergo large-scale clinical trials. It has not been included in clinical guidelines or standardized assessment protocols, making it difficult for hospitals to widely adopt or procure.

The following (Table 2) is a structured overview of the above three intelligent assessment methods: optical, vision-based, and radar-based systems, with a focus on introducing their core advantages and applicability to representative motion patterns.

### 3.4. Inertial Sensor Technology

#### 3.4.1. Mechanism

The inertial measurement unit (IMU) includes an accelerometer, a gyroscope, and a magnetometer, which can be used for posture detection, gait evaluation, and fall warning by measuring triaxial acceleration, angular velocity, and motion direction [55]. Arm and hand joint parameters are commonly measured using an IMU [56]. In recent years, more groups have utilized IMUs to measure lower limb joint parameters. IMUs have inherent drift errors that increase over time when calculating limb position or angle because of the continuous fusion of signals [57]. A series of new assessment systems that combine artificial intelligence and IMUs have been developed to increase the assessment accuracy.

#### 3.4.2. New Findings

Wearable sensor systems can estimate kinematics in any setting. Previous systems performed less accurately than OMC and required proximity to a computer and the use of proprietary software, which limited the reproducibility of experiments. Two recent studies that compared gold standard OMCS with IMUs for measuring scapular kinematics and lower extremity motion asymmetry reported that IMUs were more accurate than OMC when placed at the shoulder crest and that the IMUs produced large errors in detecting motion in the transverse plane [58, 59]. OpenSenseRT [60] is an open-source wearable system that provides real-time assessments of the kinematics of the upper and lower extremities through the use of an IMU and a portable microcontroller. Compared with gold standard OMCS, these systems are more accurate, less expensive, and easier to replicate for motion analysis in clinical, home, and free-living environments. Duan et al. [61] proposed a method for identifying five types of lower limb movements using the built-in inertial sensors of an Android smartphone. They employed fast Fourier transform for feature extraction and evaluated three machine learning classifiers—naive Bayes, K-nearest neighbor (KNN), and artificial neural networks—for activity recognition. After motion features were extracted, a subset of features was selected as a feature vector via fast Fourier transform and three supervised learning algorithms (parsimonious Bayes, K-nearest neighbor [KNN], and artificial neural networks) to classify and predict human lower limb movements. Yang et al. [62] placed IMU sensors on the torso and feet for heading angle detection during turns and proposed a hierarchical classification model that can recognize two gait tasks and four gait phases during turns, providing quantitative spatiotemporal parameters of gait. Zumaeta et al. [63] combined optical flow and inertial sensors to accurately measure the frequency of repetitive finger movements, providing a viable basis for assessing motor retardation.

#### 3.4.3. Disease Identification and Severity Assessment

A comprehensive overview of recent studies utilizing IMU-based and multisensor systems for motion assessment in neurological disorders is provided in Table 3, particularly PD and stroke. The devices range from wearable accelerometers and gyroscopes to multisensor gloves and smartphone-integrated systems. Across various studies, these technologies have proven effective in capturing gait asymmetry, freezing episodes, spasticity, and tremor characteristics. Notably, ML algorithms such as CNN, SVM, and RF were frequently employed to enhance diagnostic precision. These tools offer promising, noninvasive, and often portable methods for continuous monitoring and individualized rehabilitation planning in both clinical and home settings.

### 3.5. Robotic Feedback Technology

#### 3.5.1. Mechanism

Robot feedback technology refers to the use of sensors by robots to obtain data such as muscle activity and movement information. The data are transmitted to a computer for processing and analysis and compared with normal data, which allows posture, position, velocity, and other states to be monitored [72]. Robotic feedback techniques typically include positional feedback, force feedback, visual feedback, and acoustic feedback. A range of new assessment tools based on robotic feedback technology have been developed to provide new interventions for the assessment and treatment of abnormal movement patterns.

#### 3.5.2. New Findings

Jiang et al. [73] combined a rehabilitation robot and a motion capture system to evaluate upper limb motor function. They selected seven evaluation metrics, including ROM, shoulder girdle compensation, trunk compensation, aiming angle, movement error, length of motion ratio, and forcefulness. The validity of this assessment system was demonstrated in healthy subjects. Welwalk WW-2000, a revolutionary gait training robot that is outfitted with sensors and a markerless motion capture system, can detect abnormal hemiplegic gait patterns during robot-assisted gait training [74]. Abnormal gait patterns, including hip hiking, circumduction, and contralateral vaulting, were simulated in healthy participants.

#### 3.5.3. Disease Identification and Severity Assessment

Currently, abnormal gaits are identified using data extracted from wearable sensors, specifically pressure and inertial data. However, this approach is associated with drawbacks, such as inaccurate data and difficulties for patients in wearing these sensors, which hinder their application. Park et al. [75] developed a haptically intelligent device for elbow spasticity called the HESS. Four haptic models (HMs) were created to quantify the tactile sensation of different levels of spasticity (MASs 1, 1+, 2, and 3) based on clinical assessments of the MAS and quantitative data (position, velocity, and torque) collected from subjects with elbow spasticity. Park et al. [76] used HESS to collect data such as joint motion, calculated nine biomechanical parameters to describe the spasticity response, trained an artificial neural network to learn MAS assessment from the parameters to predict MAS scores, and concluded that the capture angle, maximum stretch speed, and maximum resistance were the most relevant parameters influencing AI decisions. The kinematic assessment of passive stretch (KAPS) is a method to assess elbow flexor and extensor spasticity following stroke; it uses a robotic exoskeleton and involves passive elbow flexion and extension over a range of 80° at five movement durations (1500, 1200, 1000, 800, and 600 ms) [77]. Seven specific parameters (peak velocity, final angle, creep [or release], interarm peak velocity difference, interarm final angle, interarm creep, and interarm pinch angle) were selected and evaluated to characterize spasticity. The shortest movement duration (600 ms) was determined to be the most helpful for identifying spasticity-related impairments. The decrease in peak velocity during passive stretching of the affected and unaffected limbs was most effective in judging whether an individual was impaired. Baur et al. [78] first applied remote 3D haptic assessment to telerehabilitation, proposing a “Beam-Me-In Strategy” robot-assisted telerehabilitation system that accurately assessed four typical abnormal movement patterns in stroke patients: a reduced active–passive ROM, resistance to passive movement, a lack of catabolic motion, and disturbances in the quality of movement. The neurorehabilitation robot was able to quantify abnormal synergistic patterns in the forearm, elbow, and shoulder joints in patients with chronic stroke [79]. The angle of the elbow joint strongly correlates with the anterior–posterior torque of the forearm in stroke patients. The forearm torque significantly varies, and abnormal cocontraction of the elbow and shoulder muscles occurs. Differences were more pronounced in the left–right direction and in the proximal–contralateral tilt direction.

## 4. Physiological and Neuromuscular Assessment Technologies

### 4.1. Multilead EMG

#### 4.1.1. Mechanism

EMG is an electrodiagnostic modality employed to assess and document the electrical impulses produced by the musculoskeletal system. When nerves or electricity stimulate muscle cells, EMG can detect the electrical potentials they produce. Signal analysis can be used to detect medical abnormalities, activation levels, or movement sequences, or it can be applied to biomechanical studies, including analyses of human and animal movement. Two types of EMG are available: surface electromyography (sEMG) and intramuscular EMG. They are more commonly to identify muscles responsible for movement and can be used to study the function of skeletal muscles and the synergistic activity between them to analyze the function of skeletal muscles in different movements, postures, and their synergistic effects [80]. sEMG is a method to collect electrical activity signals from muscle motor units through surface electrodes. Its signal is a voltage–time series obtained by capturing and analyzing the bioelectrical alterations in the neuromuscular system during various activities using surface electrodes. The amplitude is approximately 0–5000 *μ*V, and the frequency is 30–350 Hz. It can reflect neuromuscular activity to some extent and is widely used in medicine, rehabilitation, human–computer interaction, and other fields [81]. Needle EMG, which records electrical signals by inserting needles into muscles, helps us understand the activity of muscle fibers and the function of motion units, but the quality of the recorded signal is affected by a variety of factors, such as the type of electrode used and the settings of the filters and amplifiers [82].

#### 4.1.2. New Findings

sEMG has the advantages of being accurate, safe, simple, and noninvasive, and it can obtain information about muscle activity without piercing the skin [83]. It is also painless and nonirritating and has no side effects during the examination, making it relatively easy to perform and acceptable to test subjects. sEMG provides a quantitative and qualitative description of the activity of active and antagonistic muscles and changes in muscle force and tone during motor control. Time domain indicators, such as integrated EMG values and root mean square values, can provide an objective picture of the muscle activity being measured [84]. Recent advances highlight the potential of DL techniques in improving the accuracy and robustness of EMG signal decoding for myoelectric control. Both the integration of multimodal inputs and the development of realistic EMG simulation frameworks have demonstrated significant promise in addressing limitations of traditional ML approaches [85, 86]. Needle EMG can record and analyze the electrical signals generated by individual muscle fibers of the motor unit during rest and random contractions and interpret these signals to determine the function of muscle fibers and motor units. However, this system requires the insertion of needle electrodes into the muscle to accurately study the kinematic and neurophysiological activity of deep muscles, and it has a smaller test range than sEMG [82]. Two new studies have demonstrated the feasibility of applying ML and DL techniques to classify resting needle EMG discharges. By leveraging methods such as Mel-spectrogram conversion, data augmentation, feature extraction, and transfer learning, both approaches achieved high classification accuracy. These findings suggest that AI-assisted interpretation of needle EMG signals could serve as a valuable tool in clinical neurophysiology [87].

#### 4.1.3. Disease Identification and Severity Assessment

EMG technology can help identify spastic muscles, identify active and antagonistic muscles associated with tremor, and aid in botulinum toxin treatment to determine whether the injection needle is entering the target muscle. Alves et al. [88] used sEMG sensors and angiometry, which has more reliable embedded signal processing techniques, to quantify the response of passively stretched spastic muscles by measuring the tonic stretched reflex threshold, which was found to be positively correlated with the MAS. Moreover, Frenkel-Toledo et al. suggested good interassessor reliability [89]. However, these two studies still relied on extensive manual stretching to induce reflex responses [88, 89], which could lead to monotony and exhaustion for both experimenters and participants. Additionally, the intricate placement of sEMG sensors further reduces the practicality and utility of measuring spasticity in this manner [90]. Recent developments have explored the integration of EMG and kinematic data for automated detection. One study proposed an EMG-based sensor node and algorithm to detect spasticity status during normal walking [91]. This new method revealed limited antagonistic muscle coactivation in individuals with unilateral cerebral palsy. Pascual-Valdunciel et al. [92] developed a tremor detection method that combines kinematics and EMG signals that utilizes traditional ML techniques (SVM, KNN, and RF) and DL models to binarily classify pathological tremor signals without extracting features. Additionally, a wearable system incorporating IMU and sEMG sensors was used to assess upper limb motor function across 11 functional tasks. Motor abnormalities, including tremor, joint range limitations, and impaired daily activity performance, were quantified using ML-based metrics, providing a comprehensive evaluation of upper extremity function [93].

Needle EMG has been used to guide the localization of botulinum toxin injections for the treatment of spastic diagonal necks. The use of EMG enables the determination of the target muscle as well as the intensity of the screen group firing site bursts; combined with the sound of the speaker, EMG is used to determine the injection site [94]. Cesqui et al. [95] demonstrated that EMG pattern recognition methods cannot assess the kinematics of subjects suffering from neurological injuries such as stroke. They suggested that EMG signals should be analyzed in the future to gain insight into the state of muscle activity, identify possible abnormal patterns, and provide individuals timely feedback on correct muscle recruitment.

### 4.2. Ultrasound Technology

#### 4.2.1. Mechanism

Quantitative ultrasound (QUS) techniques [96], such as ultrasound strain imaging and shear wave elastography, can distinguish between normal and diseased tissues based on their echogenicity and mechanical properties. QUS is a technique that uses the propagation properties of ultrasound to assess the properties of bone and soft tissue. Quantitative information about the tested tissue can be obtained by measuring the physical properties of ultrasound, such as speed, attenuation, and scattering, as it propagates through the bone or soft tissue. QUS is less expensive than magnetic resonance imaging and can be performed on a larger group of patients (requiring less patient cooperation and with few contraindications), such as patients in medical facilities.

#### 4.2.2. New Findings

Conventional ultrasound elastography can be used to examine the dynamic elasticity of muscles. During the recording of passive dorsiflexion of a joint, the muscle ultrasound elastic modulus values corresponding to the joint angle change. Further tracing of the elastic modulus–joint angle curve and comparisons of the differences in the curve parameters E0 (elasticity value in a state of complete muscle relaxation), relaxation angle (angle of joint movement when muscle tone begins to increase), muscle thickness, and length between different populations can be used to assess biomechanical characteristics, such as the state of muscle tone [97]. Hu et al. [98] developed a novel wearable ultrasound patch that provides continuous, noninvasive 3D imaging of tissue 4 cm below the human skin surface at a spatial resolution of 0.5 mm for assessing tissue stiffness. Implementing the functionality of conventional ultrasound testing and overcoming the limitations of conventional ultrasound technology, such as single tests, in-hospital-only testing, and the need for staff manipulation, allows patients to continuously monitor their health status anywhere and at any time. Wearable A-mode ultrasound and sEMG were comprehensively compared in terms of gesture recognition and isometric muscle contraction force estimation [99]. The results indicated that A-mode US exhibited better performance in terms of gesture recognition accuracy, robustness, and discrete force estimation accuracy than sEMG, whereas sEMG outperformed US in terms of continuous force estimation accuracy and ease of use.

#### 4.2.3. Disease Identification and Severity Assessment

QUS is used to evaluate characteristics related to the mechanical properties of tissues, such as tissue morphology and stiffness, and is commonly used to assess the severity of dystonia [96]. Recent studies have demonstrated the growing potential of ultrasound elastography in assessing spasticity across various neurological conditions, particularly poststroke spasticity and multiple sclerosis. In stroke patients, strain imaging parameters of the biceps brachii significantly differed among normal, nonspastic, and spastic muscles, particularly at 90° elbow flexion and maximal extension. It indicated the viability of strain imaging in differentiating spasticity severity [100]. Two-dimensional shear wave elastography (2D-SWE) holds clinical value for the early detection of mild spasticity as well as the accurate diagnosis of severe spasticity. The sliding angle measured by 2D-SWE showed high intra- and interobserver reliability and was significantly correlated with MAS scores. Receiver operating characteristic analysis indicated that the modified sliding angle had high sensitivity and specificity in distinguishing spasticity grades, especially severe cases [101]. The combination of SWE and EMG enabled the evaluation of botulinum toxin efficacy in poststroke spastic dystonia and reveals SWE's capacity to detect intrinsic muscle remodeling [102]. Illomei et al. [103] studied the use of real-time ultrasound elastography to objectively assess the status of quadriceps muscle fibers in patients with multiple sclerosis spasticity and their changes following antispasticity treatment. A new score, the MEMS (Muscle Elasticity Multiple Sclerosis Score, range 1–5), was introduced based on the range of color coding of the elastic images, where red indicates areas of maximum strain or maximum elasticity (i.e., the softest tissue) and blue suggests no strain or no elasticity.

### 4.3. Infrared Thermal Imaging Technology

#### 4.3.1. Mechanism

Infrared thermography is a noninvasive and noncontact technique. As a specific type of RGB image, its dimensionality and computational intensity are lower than those of a normal RGB image, whereas its color signature is more abundant. The control of posture and movement includes proprioceptive mechanisms that are afferent to muscles, joints, and skin. Differences in muscle tone and strength between the active and antagonistic muscles on the affected side caused by neurological disorders result in an asymmetry of activity between the affected and healthy sides. Muscle and tissue metabolism is required during activity, resulting in altered local blood flow and local vasodilation of small arteries used to provide energy to the muscles [104]. The distribution of the skin temperatures of these affected tissues can be represented in the thermogram. Therefore, infrared thermography can be used to assess abnormal movement patterns in patients. Using the radiation difference between the object and the background environment and the radiation difference of each part of the object itself, infrared thermography can show the radiation fluctuation to capture precise postural information [105].

#### 4.3.2. New Findings

In a recent study, an infrared thermal imaging camera was used to capture thermal images of hand movements in real time, and the 3D coordinates of hand joints were extracted from the thermal images using a transformer-based spatiotemporal network [105]. The angles of finger joint movements were calculated from the coordinates obtained from infrared thermography and used to measure fine movements. The system's accuracy and stability for the finger motion angle calculation task were compared to those of the Azure Kinect system and the leap motion system, indicating that it can be used for effective and simple finger motion measurement in biomedical science. This system is expected to be used as a new method for biomedical assessment.

#### 4.3.3. Disease Identification and Severity Assessment

A recent study reported a noninvasive infrared thermography technique that uses autonomic dysfunction as a diagnostic criterion [106]. This technique was able to discriminate PD tremors from essential tremor and normal subjects. An analysis of hand thermography revealed a decrease in the mean baseline temperature in patients with PD, contributing to the early detection of PD. Moreover, infrared thermography could become an important future tool in investigation of autonomic deficiency in PD. A study examined 21 PD patients and 19 healthy controls using infrared thermographic imaging at various body sites before and after a cold stress test, calculating thermal recovery rates and analyzing their correlation with autonomic symptoms and clinical data. PD patients showed significantly reduced recovery rates, particularly at the fifth distal phalanx 10 min posttest, although no significant correlations were found between thermal parameters and autonomic measures [107]. In addition, hemiplegia affects lower limb muscle circulation and thus lowers limb temperature. Therefore, this system can also identify spastic muscles by monitoring lower limb temperature in patients with hemiplegia for corresponding treatment [108].

### 4.4. PET/SPECT Muscle Metabolism Imaging

#### 4.4.1. Mechanism

Both PET and SPECT muscle metabolism imaging involve injecting radioactive imaging agents into the body and then observing changes in muscle tissue metabolism through PET/SPECT scanning. The common imaging agent used in PET/CT is 18F-deoxyglucose (18F-FDG), and that used in SPECT/CT is technetium [99mTc] methoxyisobutylmethoxyisocyanide (99mTc-MIBI). When the imaging agent is taken up by the muscle in the body, it is metabolized to produce positrons that emit gamma radiation, which is detected by the PET/SPECT scanner, and the computer reconstructs an image of the metabolism of the muscle tissue from the digital signal.

#### 4.4.2. General Applications

SPECT/CT is commonly used for myocardial perfusion imaging and uses 99mTc-MIBI as the imaging agent to reflect changes in perfusion and local mitochondrial metabolism in muscle tissue. It has proven to be highly useful in the clinical setting. Nuclear imaging is commonly used for cardiac function tests to assess myocardial survival, understand the specifics of ischemic or infarcted myocardium, and provide an important basis for the diagnosis of coronary microcirculatory disease [109].

#### 4.4.3. Disease Identification and Severity Assessment

Based on the principles of SPECT/PET for assessing myocardial metabolism, PET/SPECT was first used to assess dystonia, which can help physicians determine the severity and extent of the responsible muscles that cause abnormal movement patterns by comparing images of the metabolic profile of normal muscle tissue with that of abnormal muscle tissue. Sung et al. [110] were the first to report the use of 18F-FDG PET/CT imaging to identify abnormal metabolism in neck muscles to determine the muscle responsible for spasticity. PET/CT images were used to assess the name and number of hypermetabolic muscles as well as the standardized uptake value maximum for each hypermetabolic muscle [111]. Furthermore, these images were used as an adjunct to botulinum toxin injections in dystonic muscles. Revuelta et al. [112] used 18F-FDG PET/CT to study hyperactivity in deep muscles and showed that PD and anterior cervical dystonia differ in their muscle activation patterns. However, 18F-FDG PET/CT is expensive, has a high radiation dose, and is not widely used. Chen et al. [113] demonstrated for the first time that 99mTc-MIBI SPECT/CT is an effective method for assessing target muscles in patients with cervical dystonia. The use of 99mTc-MIBI SPECT/CT improved the detection rate of deep muscles, visualized the distribution of affected muscles, and exhibited good sensitivity and consistency compared with EMG, providing guidance for the analysis and refinement of small muscles in the neck. A retrospective analysis using SPECT/CT scanning of the inferior cephalic oblique muscle in patients with scoliosis revealed strong concordance between EMG and SPECT/CT for the diagnosis of excitatory obliquus capitis inferior [114].

## 5. Conclusion

In summary, the latest intelligent assessment solutions have gradually moved from overall movement patterns to fine motor and muscle assessment, improving the accuracy of the assessment methods with the help of artificial intelligence and algorithms. However, their clinical applicability is still lacking. The sample size in current studies is small. The use and development of intelligent assessment systems need to consider the environment in which they are used. With the increasing demand for home rehabilitation, such as telerehabilitation, an ideal motion capture system should not only be accurate in estimating postures but also be affordable and user-friendly to facilitate manipulation by individuals with limited experience or knowledge of motion capture technology. In the clinical environment, these systems need to be easy to use for the patient or assessor, rapidly provide accurate results, overcome room lighting perspectives, etc. With innovations in technology and the optimization of assessment techniques, the future holds the promise of developing intelligent assessment tools that are more accurate and suitable for both clinical and home environments to provide more precise treatment options to patients.

## Figures and Tables

**Table 1:** Vision-based assessment studies in neurological disorders.

**Authors**	**Method**	**Application**	**Key features**	**Key findings**
Rupprechter et al. [43]	CV-based gait analysis	PD	Speed, swing, roughness, postural control	Strong correlation with UPDRSIII; reduced stride variability in PD
Liu et al. [44]	Eulerian video magnification + CV	PD tremor	Amplification of subtle tremor	Accurate assessment of hand, leg, jaw, and postural tremors
Guo et al. [45]	CV + SVM for finger tapping test	PD detection and monitoring	Amplitude, speed, rhythm; UPDRS score prediction	Enables early PD detection and treatment comparison
Hyppönen et al. [8]	Video-based automatic myoclonus rating	Myoclonus	Jerk detection and scale development	Moderate correlation with Unified Myoclonus Rating Scale

**Table 2:** A structured overview of the above three intelligent assessment methods.

**Abnormal movement pattern**	**Device type**	**Core advantages**	**Limitations**	**Patient setup time**
PD tremor monitoring	Optical (e.g., Vicon)	> 200-Hz temporal resolution; submillimeter spatial precision Gold standard for lab-based tremor quantification	High cost Marker attachment Lab-dependent environment	> 30 min
	Visual (e.g., Azure Kinect)	Arker-free; moderate cost	Light-dependent; centimeter-level precision; limited microtremor capture	< 10 min
	Radar	Micromotion sensitivity (penetrates clothing); contactless (5-min deployment); enables home-based continuous monitoring	Remote tracking	5 min

Gait analysis	Optical	Provides joint torque and full biomechanical parameters Lab-grade high precision	Space-constrained; complex operation Requires professionals	> 30 min
	Visual	Moderate cost; supports spatiotemporal parameters (speed/stride length)	Environmental light affects stability	< 10 min
	Radar	Wall-penetrating monitoring; omnidirectional activity tracking; suitable for ward/home environments	Inability to output anatomical details (e.g., joint angles); limited spatial resolution	5 min

**Table 3:** Overview of IMU-based assessment in neurological disorders.

**Authors**	**Device type**	**Target population**	**Main assessment**	**Key findings**
Lee et al. [64]	Wearable (3-axis accelerometer + gyroscope)	Hemiplegic patients	Gait symmetry analysis	Normal gait: Regular, fewer steps; L/R axis movement identifies hemiplegia
Kim et al. [65]	Android phone (3-axis accelerometer + gyroscope)	PD patients (freezing of gait)	Remote monitoring of freezing duration/episodes	CNN performed best; pocket placement improved usability
Abdollahi et al. [66]	4 IMUs + pressure platform	Poststroke patients	Turning and walking kinematics	Increased trunk/sacral lateral flexion and double support in the stroke group
Lin et al. [67]	Multisensor glove system (IMU + pressure sensors)	Patients with mild to moderate finger spasticity	Spasm detection during voluntary hand motion	Angular velocity/variability features distinguish spasticity (*p* < 0.05)
Amit et al. [68]	Anesthesia monitoring glove (caregiver-worn IMU + pressure sensors)	Spasticity patients	Monitoring during anesthesia induction	Differentiates neural vs. nonneural resistance; quantifies dynamic spasticity
Jeon et al. [69]	Wristwatch-type device (accelerometer + gyroscope)	PD patients	Automatic tremor scoring	Amplitude, regularity, frequency features + SVM/RF/KNN classifiers
Sigcha et al. [70]	Consumer smartwatch (built-in accelerometer)	Early-stage PD patients	Tremor amplitude and vibration monitoring	Multitask CNN accurately detects early tremor
Rabelo et al. [71]	Inertial+ EMG sensors	PD vs. healthy elderly	Wrist extension: Motion and muscle activity	Mean/std error differentiates PD from controls

## Data Availability

No underlying data was collected or produced in this study.

## References

[1] Sethi A., Ting J., Allen M., Clark W., Weber D. (2020). Advances in Motion and Electromyography Based Wearable Technology for Upper Extremity Function Rehabilitation: A Review. *Journal of Hand Therapy*.

[2] Hssayeni M. D., Jimenez-Shahed J., Burack M. A., Ghoraani B. (2019). Wearable Sensors for Estimation of Parkinsonian Tremor Severity During Free Body Movements. *Sensors (Basel)*.

[3] Mascia M. M., Orofino G., Cimino P., Cadeddu G., Ercoli T., Defazio G. (2022). Writing Tremor in Parkinson’s Disease: Frequency and Associated Clinical Features. *Journal of Neural Transmission (Vienna)*.

[4] Platz T. (2019). Evidence-Based Guidelines and Clinical Pathways in Stroke Rehabilitation-an International Perspective. *Frontiers in Neurology*.

[5] Bao Y. J., Hong R. H., Qin W. T., Wu Z., Song Y. P., Jin L. J. (2024). Intelligent Assessment of Abnormal Movements in Neurological Disorders: An Update [abstract]. *Movement Disorders*.

[6] Ansari N. N., Naghdi S., Hasson S., Azarsa M. H., Azarnia S. (2008). The Modified Tardieu Scale for the Measurement of Elbow Flexor Spasticity in Adult Patients With Hemiplegia. *Brain Injury*.

[7] Patrick E., Ada L. (2006). The Tardieu Scale Differentiates Contracture From Spasticity Whereas the Ashworth Scale Is Confounded by It. *Clinical Rehabilitation*.

[8] Thenganatt M. A., Louis E. D. (2012). Distinguishing Essential Tremor From Parkinson’s Disease: Bedside Tests and Laboratory Evaluations. *Expert Review of Neurotherapeutics*.

[9] Celik Y., Stuart S., Woo W. L., Godfrey A. (2021). Gait Analysis in Neurological Populations: Progression in the Use of Wearables. *Medical Engineering & Physics*.

[10] Howard I. M., Patel A. T. (2023). Spasticity Evaluation and Management Tools. *Muscle & Nerve*.

[11] Elble R. J., Ondo W. (2022). Tremor Rating Scales and Laboratory Tools for Assessing Tremor. *Journal of the Neurological Sciences*.

[12] Hyppönen J., Hakala A., Annala K. (2020). Automatic Assessment of the Myoclonus Severity From Videos Recorded According to Standardized Unified Myoclonus Rating Scale Protocol and Using Human Pose and Body Movement Analysis. *Seizure*.

[13] Herda L., Fua P., Plänkers R., Boulic R., Thalmann D. (2001). Using Skeleton-Based Tracking to Increase the Reliability of Optical Motion Capture. *Human Movement Science*.

[14] McHugh B., Akhbari B., Morton A. M., Moore D. C., Crisco J. J. (2021). Optical Motion Capture Accuracy Is Task-Dependent in Assessing Wrist Motion. *Journal of Biomechanics*.

[15] Smirnova V., Khamatnurova R., Kharin N., Yaikova E., Baltina T., Sachenkov O. (2022). The Automatization of the Gait Analysis by the Vicon Video System: A Pilot Study. *Sensors*.

[16] Lee K. D., Park H. S. (2022). Real-Time Motion Analysis System Using Low-Cost Web Cameras and Wearable Skin Markers. *Frontiers in Bioengineering and Biotechnology*.

[17] Wang L., Li Y., Xiong F., Zhang W. (2021). Gait Recognition Using Optical Motion Capture: A Decision Fusion Based Method. *Sensors*.

[18] Fonk R., Schneeweiss S., Simon U., Engelhardt L. (2021). Hand Motion Capture From a 3D Leap Motion Controller for a Musculoskeletal Dynamic Simulation. *Sensors*.

[19] Klöpfer-Krämer I., Brand A., Wackerle H., Müßig J., Kröger I., Augat P. (2020). Gait Analysis - Available Platforms for Outcome Assessment. *Injury*.

[20] Litinas K., Roenigk K. L., Daly J. J. (2022). Thigh and Shank, Kinetic and Potential Energies During Gait Swing Phase in Healthy Adults and Stroke Survivors. *Brain Sciences*.

[21] Xie Q., Sheng B., Huang J., Zhang Q., Zhang Y. (2022). A Pilot Study of Compensatory Strategies for Reach-to-Grasp-Pen in Patients With Stroke. *Applied Bionics and Biomechanics*.

[22] Johansson G. M., Grip H., Levin M. F., Häger C. K. (2017). The Added Value of Kinematic Evaluation of the Timed Finger-to-Nose Test in Persons Post-Stroke. *Journal of Neuroengineering and Rehabilitation*.

[23] Khwaounjoo P., Singh G., Grenfell S. (2022). Non-Contact Hand Movement Analysis for Optimal Configuration of Smart Sensors to Capture Parkinson’s Disease Hand Tremor. *Sensors (Basel)*.

[24] Colyer S. L., Evans M., Cosker D. P., Salo A. I. T. (2018). A Review of the Evolution of Vision-Based Motion Analysis and the Integration of Advanced Computer Vision Methods Towards Developing a Markerless System. *Sports Medicine-Open*.

[25] Ripic Z., Nienhuis M., Signorile J. F., Best T. M., Jacobs K. A., Eltoukhy M. (2023). A Comparison of Three-Dimensional Kinematics Between Markerless and Marker-Based Motion Capture in Overground Gait. *Journal of Biomechanics*.

[26] Dilek E., Dener M. (2023). Computer Vision Applications in Intelligent Transportation Systems: A Survey. *Sensors (Basel)*.

[27] van der Velden B. H. M., Kuijf H. J., Gilhuijs K. G. A., Viergever M. A. (2022). Explainable Artificial Intelligence (XAI) in Deep Learning-Based Medical Image Analysis. *Medical Image Analysis*.

[28] Kanko R. M., Laende E. K., Davis E. M., Selbie W. S., Deluzio K. J. (2021). Concurrent Assessment of Gait Kinematics Using Marker-Based and Markerless Motion Capture. *Journal of Biomechanics*.

[29] Zhao Y., Li J., Wang X. (2022). A Lightweight Pose Sensing Scheme for Contactless Abnormal Gait Behavior Measurement. *Sensors*.

[30] Young F., Mason R., Morris R., Stuart S., Godfrey A. (2023). Internet-of-Things-Enabled Markerless Running Gait Assessment From a Single Smartphone Camera. *Sensors (Basel)*.

[31] Kim B., Neville C. (2023). Accuracy and Feasibility of a Novel Fine Hand Motor Skill Assessment Using Computer Vision Object Tracking. *Scientific Reports*.

[32] Faity G., Mottet D., Froger J. (2022). Validity and Reliability of Kinect v2 for Quantifying Upper Body Kinematics During Seated Reaching. *Sensors*.

[33] Eltoukhy M., Oh J., Kuenze C., Signorile J. (2017). Improved Kinect-Based Spatiotemporal and Kinematic Treadmill Gait Assessment. *Gait and Posture*.

[34] Knippenberg E., Verbrugghe J., Lamers I., Palmaers S., Timmermans A., Spooren A. (2017). Markerless Motion Capture Systems as Training Device in Neurological Rehabilitation: A Systematic Review of Their Use, Application, Target Population and Efficacy. *Journal of Neuroengineering and Rehabilitation*.

[35] Bakhti K. K. A., Laffont I., Muthalib M., Froger J., Mottet D. (2018). Kinect-Based Assessment of Proximal Arm Non-Use After a Stroke. *Journal of Neuroengineering and Rehabilitation*.

[36] Hong R., Zhang T., Zhang Z. (2022). A Summary Index Derived From Kinect to Evaluate Postural Abnormalities Severity in Parkinson’s Disease Patients. *Npj Parkinson’s Disease*.

[37] Zhang Z., Hong R., Lin A. (2021). Automated and Accurate Assessment for Postural Abnormalities in Patients With Parkinson’s Disease Based on Kinect and Machine Learning. *Journal of Neuroengineering and Rehabilitation*.

[38] Wu Z., Hong R., Li S. (2022). Technology-Based Therapy-Response Evaluation of Axial Motor Symptoms Under Daily Drug Regimen of Patients With Parkinson’s Disease. *Frontiers in Aging Neuroscience*.

[39] Steffensen E. A., Magalhães F., Knarr B. A., Kingston D. C. (2023). Comparison of Markerless and Marker-Based Motion Capture of Gait Kinematics in Individuals With Cerebral Palsy and Chronic Stroke: A Case Study Series.

[40] Du H., Henry P., Ren X. (2011). Interactive 3D Modeling of Indoor Environments With a Consumer Depth Camera. *Proceedings of the 13th International Conference on Ubiquitous Computing*.

[41] Zhu Y., Lu W., Gan W., Hou W. (2021). A Contactless Method to Measure Real-Time Finger Motion Using Depth-Based Pose Estimation. *Computers in Biology and Medicine*.

[42] Kincaid C. J., Vaterlaus A. C., Stanford N. R., Charles S. K. (2019). Frequency Response of the Leap Motion Controller and Its Suitability for Measuring Tremor. *Medical Engineering & Physics*.

[43] Rupprechter S., Morinan G., Peng Y. (2021). A Clinically Interpretable Computer-Vision Based Method for Quantifying Gait in Parkinson’s Disease. *Sensors*.

[44] Liu W., Lin X., Chen X. (2023). Vision-Based Estimation of MDS-UPDRS Scores for Quantifying Parkinson’s Disease Tremor Severity. *Medical Image Analysis*.

[45] Guo Z., Zeng W., Yu T. (2022). Vision-Based Finger Tapping Test in Patients With Parkinson’s Disease via Spatial-Temporal 3D Hand Pose Estimation. *IEEE Journal of Biomedical and Health Informatics*.

[46] Zhang Z., Pouliquen P. O., Waxman A., Andreou A. G. (2007). Acoustic Micro-Doppler Radar for Human Gait Imaging. *Journal of the Acoustical Society of America*.

[47] Seifert A. K., Grimmer M., Zoubir A. M. (2021). Doppler Radar for the Extraction of Biomechanical Parameters in Gait Analysis. *IEEE Journal of Biomedical and Health Informatics*.

[48] Seifert A. K., Amin M. G., Zoubir A. M. (2019). Toward Unobtrusive In-Home Gait Analysis Based on Radar Micro-Doppler Signatures. *IEEE Transactions on Biomedical Engineering*.

[49] Jiang X., Zhang Y., Yang Q., Deng B., Wang H. (2020). Millimeter-Wave Array Radar-Based Human Gait Recognition Using Multi-Channel Three-Dimensional Convolutional Neural Network. *Sensors (Basel)*.

[50] Soubra R., Mourad-Chehade F., Chkeir A. (2023). Automation of the Timed Up and Go Test Using a Doppler Radar System for Gait and Balance Analysis in Elderly People. *Journal of Healthcare Engineering*.

[51] Liang X., Deng J., Zhang H., Gulliver T. A. (2018). Ultra-Wideband Impulse Radar Through-Wall Detection of Vital Signs. *Scientific Reports*.

[52] Yim D., Lee W. H., Kim J. I. (2019). Quantified Activity Measurement for Medical Use in Movement Disorders Through IR-UWB Radar Sensor. *Sensors*.

[53] Na J. Y., Lee W. H., Lim Y. H., Cho S. H., Cho S. H., Park H. K. (2022). Early Screening Tool for Developmental Delay in Infancy: Quantified Assessment of Movement Asymmetry Using IR-UWB Radar. *Frontiers in Pediatrics*.

[54] Zilani T. A., Al-Turjman F., Khan M. B., Zhao N., Yang X. (2020). Monitoring Movements of Ataxia Patient by Using UWB Technology. *Sensors*.

[55] Faisal A. I., Majumder S., Mondal T., Cowan D., Naseh S., Deen M. J. (2019). Monitoring Methods of Human Body Joints: State-of-the-Art and Research Challenges. *Sensors (Basel)*.

[56] Wang Q., Markopoulos P., Yu B., Chen W., Timmermans A. (2017). Interactive Wearable Systems for Upper Body Rehabilitation: A Systematic Review. *Journal of Neuroengineering and Rehabilitation*.

[57] Ricci L., Taffoni F., Formica D. (2016). On the Orientation Error of IMU: Investigating Static and Dynamic Accuracy Targeting Human Motion. *PLoS One*.

[58] Friesen K. B., Sigurdson A., Lang A. E. (2023). Comparison of Scapular Kinematics From Optical Motion Capture and Inertial Measurement Units During a Work-Related and Functional Task Protocol. *Medical & Biological Engineering & Computing*.

[59] Teufl W., Miezal M., Taetz B., Fröhlich M., Bleser G. (2022). P 052 - Detection of Lower Extremity Asymmetries in Slow and Dynamic Bilateral Tasks. Inertial Sensor System vs Optical Motion Capture System. *Gait and Posture*.

[60] Slade P., Habib A., Hicks J. L., Delp S. L. (2022). An Open-Source and Wearable System for Measuring 3D Human Motion in Real-Time. *IEEE Transactions on Biomedical Engineering*.

[61] Duan L. T., Lawo M., Wang Z. G., Wang H. Y. (2022). Human Lower Limb Motion Capture and Recognition Based on Smartphones. *Sensors*.

[62] Yang Y., Chen L., Pang J., Huang X., Meng L., Ming D. (2022). Validation of a Spatiotemporal Gait Model Using Inertial Measurement Units for Early-Stage Parkinson’s Disease Detection During Turns. *IEEE Transactions on Biomedical Engineering*.

[63] Zumaeta K., Romero S. E., Torres E. (2021). Combining Inertial Sensors and Optical Flow to Assess Finger Movements: Pilot Study for Telehealth Applications. *Proceedings of the 2021 43rd Annual International Conference of the IEEE Engineering in Medicine & Biology Society (EMBC)*.

[64] Lee J., Park S., Shin H. (2018). Detection of Hemiplegic Walking Using a Wearable Inertia Sensing Device. *Sensors (Basel)*.

[65] Kim H. B., Lee H. J., Lee W. W. (2018). Validation of Freezing-of-Gait Monitoring Using Smartphone. *Telemedicine Journal and E-Health*.

[66] Abdollahi M., Kuber P. M., Hoang C., Shiraishi M., Soangra R., Rashedi E. (2021). Kinematic Assessment of Turning and Walking Tasks Among Stroke Survivors by Employing Wearable Sensors and Pressure Platform. *Proceedings of the 2021 43rd Annual International Conference of the IEEE Engineering in Medicine & Biology Society (EMBC)*.

[67] Lin B. S., Lee I. J., Hsiao P. C. (2022). Design of a Multi-Sensor System for Exploring the Relation Between Finger Spasticity and Voluntary Movement in Patients With Stroke. *Sensors (Basel)*.

[68] Amit M., Yalcin C., Liu J., Skalsky A. J., Garudadri H., Ng T. N. (2022). Multimodal Assessment of Spasticity Using a Point-of-Care Instrumented Glove to Separate Neural and Biomechanical Contributions. *iScience*.

[69] Jeon H., Lee W., Park H. (2017). Automatic Classification of Tremor Severity in Parkinson’s Disease Using a Wearable Device. *Sensors*.

[70] Sigcha L., Pavón I., Costa N. (2021). Automatic Resting Tremor Assessment in Parkinson’s Disease Using Smartwatches and Multitask Convolutional Neural Networks. *Sensors*.

[71] Rabelo A. G., Neves L. P., Paixão A. P. S. (2017). Objective Assessment of Bradykinesia Estimated From the Wrist Extension in Older Adults and Patients With Parkinson’s Disease. *Annals of Biomedical Engineering*.

[72] Reyes F., Niedzwecki C., Gaebler-Spira D. (2020). Technological Advancements in Cerebral Palsy Rehabilitation. *Physical Medicine and Rehabilitation Clinics of North America*.

[73] Jiang J., Guo S., Zhang L., Sun Q. (2022). Motor Ability Evaluation of the Upper Extremity With Point-to-Point Training Movement Based on End-Effector Robot-Assisted Training System. *Journal of Healthcare Engineering*.

[74] Imoto D., Hirano S., Mukaino M., Saitoh E., Otaka Y. (2022). A Novel Gait Analysis System for Detecting Abnormal Hemiparetic Gait Patterns During Robot-Assisted Gait Training: A Criterion Validity Study Among Healthy Adults. *Frontiers in Neurorobotics*.

[75] Park H. S., Kim J., Damiano D. L. (2012). Development of a Haptic Elbow Spasticity Simulator (HESS) for Improving Accuracy and Reliability of Clinical Assessment of Spasticity. *IEEE Transactions on Neural Systems and Rehabilitation Engineering*.

[76] Park J. H., Kim Y., Lee K. J. (2019). Artificial Neural Network Learns Clinical Assessment of Spasticity in Modified Ashworth Scale. *Archives of Physical Medicine and Rehabilitation*.

[77] Centen A., Lowrey C. R., Scott S. H., Yeh T.-T., Mochizuki G. (2017). KAPS (Kinematic Assessment of Passive Stretch): A Tool to Assess Elbow Flexor and Extensor Spasticity After Stroke Using a Robotic Exoskeleton. *Journal of Neuroengineering and Rehabilitation*.

[78] Baur K., Rohrbach N., Hermsdörfer J., Riener R., Klamroth-Marganska V. (2019). The “Beam-Me-In Strategy” - Remote Haptic Therapist-Patient Interaction With Two Exoskeletons for Stroke Therapy. *Journal of Neuroengineering and Rehabilitation*.

[79] Kung P. C., Lin C. C., Ju M. S. (2010). Neuro-Rehabilitation Robot-Assisted Assessments of Synergy Patterns of Forearm, Elbow and Shoulder Joints in Chronic Stroke Patients. *Clinical Biomechanics (Bristol, Avon)*.

[80] Labanca L., Mosca M., Ghislieri M., Agostini V., Knaflitz M., Benedetti M. G. (2021). Muscle Activations During Functional Tasks in Individuals With Chronic Ankle Instability: A Systematic Review of Electromyographical Studies. *Gait and Posture*.

[81] Farina D., Merletti R., Enoka R. M. (2014). The Extraction of Neural Strategies From the Surface EMG: An Update. *Journal of Applied Physiology*.

[82] Rubin D. I. (2019). Needle Electromyography: Basic Concepts. *Handbook of Clinical Neurology*.

[83] Yu Z., Zhao J., Wang Y., He L., Wang S. (2021). Surface EMG-Based Instantaneous Hand Gesture Recognition Using Convolutional Neural Network With the Transfer Learning Method. *Sensors (Basel)*.

[84] Hu B., Zhang X., Mu J., Wu M., Wang Y. (2018). Spasticity Assessment Based on the Hilbert-Huang Transform Marginal Spectrum Entropy and the Root Mean Square of Surface Electromyography Signals: A Preliminary Study. *Biomedical Engineering Online*.

[85] Maksymenko K., Clarke A. K., Mendez Guerra I., Deslauriers-Gauthier S., Farina D. (2023). A Myoelectric Digital Twin for Fast and Realistic Modelling in Deep Learning. *Communications*.

[86] Wang W., Chen B., Xia P., Hu J., Peng Y. (2018). Sensor Fusion for Myoelectric Control Based on Deep Learning With Recurrent Convolutional Neural Networks. *Artificial Organs*.

[87] Hubers D., Potters W., Paalvast O. (2023). Artificial Intelligence-Based Classification of Motor Unit Action Potentials in Real-World Needle EMG Recordings. *Clinical Neurophysiology*.

[88] Alves C. M., Rezende A. R., Marques I. A., Martins Naves E. L. (2021). SpES: A New Portable Device for Objective Assessment of Hypertonia in Clinical Practice. *Computers in Biology and Medicine*.

[89] Frenkel-Toledo S., Solomon J. M., Shah A. (2021). Tonic Stretch Reflex Threshold as a Measure of Spasticity After Stroke: Reliability, Minimal Detectable Change and Responsiveness. *Clinical Neurophysiology*.

[90] Yu S., Chen Y., Cai Q., Ma K., Zheng H., Xie L. (2020). A Novel Quantitative Spasticity Evaluation Method Based on Surface Electromyogram Signals and Adaptive Neuro Fuzzy Inference System. *Frontiers in Neuroscience*.

[91] Misgeld B. J., Luken M., Heitzmann D., Wolf S. I., Leonhardt S. (2016). Body-Sensor-Network-Based Spasticity Detection. *IEEE Journal of Biomedical Health Informatics*.

[92] Pascual-Valdunciel A., Lopo-Martínez V., Beltrán-Carrero A. J. (2023). Classification of Kinematic and Electromyographic Signals Associated With Pathological Tremor Using Machine and Deep Learning. *Entropy (Basel)*.

[93] Li Y., Zhang X., Gong Y., Cheng Y., Gao X., Chen X. (2017). Motor Function Evaluation of Hemiplegic Upper-Extremities Using Data Fusion From Wearable Inertial and Surface EMG Sensors. *Sensors*.

[94] Wu C., Xue F., Chang W. (2016). Botulinum Toxin Type A With or Without Needle Electromyographic Guidance in Patients With Cervical Dystonia. *Springerplus*.

[95] Cesqui B., Tropea P., Micera S., Krebs H. I. (2013). EMG-Based Pattern Recognition Approach in Post Stroke Robot-Aided Rehabilitation: A Feasibility Study. *Journal of Neuroengineering and Rehabilitation*.

[96] Tran A., Gao J. (2021). Quantitative Ultrasound to Assess Skeletal Muscles in Post Stroke Spasticity. *Journal of Central Nervous System Disease*.

[97] Snoj Ž., Wu C. H., Taljanovic M. S., Dumić-Čule I., Drakonaki E. E., Klauser A. S. (2020). Ultrasound Elastography in Musculoskeletal Radiology: Past, Present, and Future. *Seminars in Musculoskeletal Radiology*.

[98] Hu H., Ma Y., Gao X. (2023). Stretchable Ultrasonic Arrays for the Three-Dimensional Mapping of the Modulus of Deep Tissue. *Nature Biomedical Engineering*.

[99] Yang X., Yan J., Liu H. (2020). Comparative Analysis of Wearable A-Mode Ultrasound and sEMG for Muscle-Computer Interface. *IEEE Transactions on Biomedical Engineering*.

[100] Gao J., Chen J., O'Dell M. (2018). Ultrasound Strain Imaging to Assess the Biceps Brachii Muscle in Chronic Poststroke Spasticity. *Journal of Ultrasound in Medicine*.

[101] Cao J., Xiao Y., Qiu W. (2022). Reliability and Diagnostic Accuracy of Corrected Slack Angle Derived From 2D-SWE in Quantitating Muscle Spasticity of Stroke Patients. *Journal of Neuroengineering and Rehabilitation*.

[102] Campanella W., Corazza A., Puce L. (2022). Shear Wave Elastography Combined With Electromyography to Assess the Effect of Botulinum Toxin on Spastic Dystonia Following Stroke: A Pilot Study. *Frontiers in Neurology*.

[103] Illomei G., Spinicci G., Locci E., Marrosu M. G. (2017). Muscle Elastography: A New Imaging Technique for Multiple Sclerosis Spasticity Measurement. *Neurological Sciences*.

[104] Merla A., Romani G. L. (2006). Functional Infrared Imaging in Medicine: A Quantitative Diagnostic Approach. *Proceedings of the 2006 international conference of the IEEE engineering in medicine and biology society*.

[105] Zhu Y., Guo C. (2023). A Hand Motion Capture Method Based on Infrared Thermography for Measuring Fine Motor Skills in Biomedicine. *Artificial Intelligence in Medicine*.

[106] Anbalagan B., Karnam Anantha S., Arjunan S., Balasubramanian V., Murugesan M., Kalpana R. (2022). A Non-Invasive IR Sensor Technique to Differentiate Parkinson’s Disease From Other Neurological Disorders Using Autonomic Dysfunction as Diagnostic Criterion. *Sensors*.

[107] Purup M. M., Knudsen K., Karlsson P., Terkelsen A. J., Borghammer P. (2020). Skin Temperature in Parkinson’s Disease Measured by Infrared Thermography. *Parkinson’s Disease*.

[108] Alfieri F. M., Massaro A. R., Filippo T. R., Portes L. A., Battistella L. R. (2017). Evaluation of Body Temperature in Individuals With Stroke. *NeuroRehabilitation*.

[109] Braat S. H. (1991). 99mTc Myocardial Perfusion Imaging. *Current Opinion in Radiology*.

[110] Sung D. H., Choi J. Y., Kim D. H. (2007). Localization of Dystonic Muscles With ^18^F-FDG PET/CT in Idiopathic Cervical Dystonia. *Journal of Nuclear Medicine*.

[111] Kwon H. R., Lee H., Sung D. H., Choi J. Y. (2022). Therapeutic Efficacy and Prediction of ^18^F-FDG PET/CT-Assisted Botulinum Toxin Therapy in Patients With Idiopathic Cervical Dystonia. *Clinical Nuclear Medicine*.

[112] Revuelta G. J., Montilla J., Benatar M. (2014). An ^18^F-FDG PET Study of Cervical Muscle in Parkinsonian Anterocollis. *Journal of the Neurological Sciences*.

[113] Chen S., Issa M. D., Wang C. (2020). [^99m^Tc]MIBI SPECT/CT for Identifying Dystonic Muscles in Patients With Primary Cervical Dystonia. *Molecular Imaging and Biology*.

[114] Su J., Hu Y., Djibo I. M. (2022). Pivotal Role of Obliquus Capitis Inferior in Torticaput Revealed by Single-Photon Emission Computed Tomography. *Journal of Neural Transmission (Vienna)*.

